# Evaluating Fecal Indicator and Pathogen Relationships in Sewage Impacted Surface Waters to Blend with Reclaimed Water for Potable Reuse in North Carolina

**DOI:** 10.3390/pathogens10121603

**Published:** 2021-12-09

**Authors:** Emily S. Bailey, Margret Hopkins, Lisa Casanova, Mark D. Sobsey

**Affiliations:** 1 Department of Environmental Sciences and Engineering, Gillings School of Global Public Health, The University of North Carolina at Chapel Hill, Chapel Hill, NC 27599, USA; margaretfhopkins@gmail.com (M.H.); mark_sobsey@unc.edu (M.D.S.); 2 Division of Environmental Health, Georgia State University, Atlanta, GA 30303, USA; lcasanova@gsu.edu

**Keywords:** pathogens, reclaimed water, reuse, surface water

## Abstract

Surface waters used for drinking water supply often receive upstream wastewater effluent inputs, resulting in de facto wastewater reuse for drinking water and recreation. As populations grow, demands on water supplies increase. As this trend continues, it creates the need to understand the risks associated with such reuse. In North Carolina, potable reuse has been proposed as a combination of at least 80% surface water with up to 20% tertiary-treated, dual-disinfected, reclaimed wastewater, which is then stored for 5 days and further treated using conventional drinking water treatment methods. The state of North Carolina has set standards for both intake surface water and for the reclaimed water produced by wastewater utilities, using indicator microorganisms to measure compliance. The goal of this study was to quantify fecal indicator microorganisms, specifically *E. coli*, coliphages, and *C. perfringens* as well as key pathogens, specifically *Salmonella* spp. bacteria, adenoviruses, noroviruses, and the protozoan parasites *Cryptosporidium and Giardia*, in two types of water representing potential candidates for potable reuse in North Carolina, (1) run of river surface water and (2) sewage-impacted surface waters, with the purpose of determining if there are predictive relationships between these two microorganism groups that support microbial indicator reliability.

## 1. Introduction

Surface waters used for drinking water supply often receive upstream wastewater effluent inputs, resulting in de facto wastewater reuse for drinking water and recreation. As populations grow, demands on water supplies increase. With growing climate change, this trend will probably continue, creating a need to quantify the microbial health risks associated with such de facto reuse [1,2,3].

Even highly treated and disinfected sewage effluents discharged to surface waters used as drinking water sources have the potential for health-related microbial risks from pathogens such as enteric viruses, including illness and potential mortality [1,4,5,6,7]. Current monitoring of the microbial quality of water and wastewater for legislated state or federal regulations is rarely based on the analysis of pathogens and instead involves the monitoring of fecal indicator microorganisms.

As regulated potable reuse of surface water/wastewater effluent blends moves forward, North Carolina has adopted the practice of using fecal indicator organisms for assessing water quality. In North Carolina, potable reuse has been defined as a combination of at least 80% surface water with up to 20% tertiary-treated, dual-disinfected, reclaimed wastewater, which is then stored for five (5) days and treated using conventional drinking water treatment methods. The state of North Carolina also has standards for both intake surface water and for the reclaimed water produced by wastewater utilities, using indicator microorganisms to measure compliance. In North Carolina, run of river (or flowing stream) systems used as source water for drinking water supply must have <300–500 fecal coliforms or *E. coli* per 100 mL depending on off-stream storage (0.5 to 4 h) [8]. These source waters must also have a minimum of five (5) days of off-stream pre-treatment/storage to maintain raw water quality and avoid plant influent water variations. North Carolina (NC) reclaimed water standards for potable ruse, referred to as type 2 reclaimed water, specify microbial reductions of 6 log_10_ for *E. coli* as a surrogate for pathogenic bacteria, 5 log_10_ for coliphages as surrogates for pathogenic enteric viruses, and 4 log_10_ for *Clostridium perfringens*, primarily their spores, as a surrogate for protozoan pathogens. 

As is common practice, the NC regulations use indicator organisms for the water quality standards as required compliance assessments for the components of these blended waters. However, it has been demonstrated that fecal indicator bacteria (FIB), and in particular coliform bacteria, do not always predict or correlate with the concentrations of pathogens in water or wastewater. This may be due to the greater susceptibility of most FIB to chemical disinfection compared to pathogens such as viruses and protozoan parasites [9,10,11,12,13,14]. Microorganisms proposed as additional indicators for the presence and concentrations of more resistant fecal pathogens in environmental, drinking and wastewater include *Enterococcus* spp. [12,13], *C. perfringens* [13], and fecal indicator viruses such as coliphages [11,14].

However, there is a need for further study of the occurrence and concentrations of alternative fecal indicators as well as enteric pathogens and the relationships between them. The goal of this study was to quantify fecal indicator microorganisms (total coliforms, *E. coli*, *C. perfringens*, and coliphages), key pathogens (*Cryptosporidium*, *Giardia*, *Salmonella* spp., adenoviruses, and noroviruses), and their relationships in surface waters allowed for potable reuse. Two types of surface waters that are candidates for blended potable reuse in North Carolina were evaluated: (1) run of river surface water and (2) surface waters receiving effluent discharges.

## 2. Results

### 2.1. Microbiological Quality

In reclaimed water samples, *E. coli* and *enterococci* bacteria were detected on average at levels of 1.12 MPN per 100 mL and 0 MPN per 100 mL, while in surface waters these bacteria were detected at levels of 2.12 × 10^2^ MPN per 100 mL and 1.98 × 10^1^ MPN per 100 mL, respectively. The concentrations are approximately 100-fold higher for both *E. coli* and *enterococci* in surface waters than in reclaimed water. For the pathogenic bacteria, *Salmonella* spp. was detected at concentrations of 0.14 MPN per 100 mL and 3.68 × 10^2^ MPN per 100 mL in reclaimed and surface waters, respectively. As with *E. coli*, there was about a 100-fold greater concentration of *Salmonella* spp. in surface waters than in reclaimed waters. However, the concentrations of *Salmonella* spp. detected in surface waters and reclaimed waters were not significantly different, with a *p*-value of 0.81.

For indicator viruses, very low levels were detected in reclaimed water samples at concentrations of 1.17, 1.29, and 1.62 PFU per 100 mL for somatic, F+, and total coliphages, respectively. In surface waters, the average concentrations of these viruses were 2.44 × 10^1^ PFU for somatic coliphages, 1.5 × 10^0^ for F+ coliphages, and 2.48 × 10^1^ for total coliphages. There are approximately 10-fold concentration differences between surface (higher concentrations) and reclaimed waters (lower concentrations) for somatic and total coliphages. However, there were similar concentrations of F+ coliphages in these two categories of water.

For the virus pathogens, no noroviruses were detected in surface waters, but, in reclaimed waters, the average concentrations were 1.73 GEC per 100 mL. Adenoviruses were detected at relatively high levels in both water types, with average concentrations in reclaimed water of 5.26 × 10^2^ GEC per 100 mL and concentrations in surface water of 1.44 × 10^4^ GEC per 100 mL; these concentrations were not significantly different (*p*-value: 0.01).

As with the other indicator organisms, low levels of *C. perfringens* were detected in reclaimed water samples, with average concentrations of 1.10 PFU per 100 mL and 1.15 PFU per 100 mL for spores and vegetative cells plus spores, respectively. In surface waters, concentrations of *C. perfringens* were 7.17 × 10^1^ CFU/100 mL for spores, and 7.99 × 10^1^ CFU/100 mL for vegetative cells plus spores, an approximately 10-fold greater concentration over reclaimed water. For *Cryptosporidium* and *Giardia*, average concentrations in reclaimed water were 0.17 oocysts per 100 mL and 0.06 cysts per 100 mL, respectively, while average concentrations in surface water were 1.18 oocysts per 100 mL and 0.26 cysts per 100 mL, respectively. Similar to the other microorganisms examined, protozoan parasites’ concentrations in surface water were approximately 10-fold greater than those in reclaimed water. For *Cryptosporidium*, the difference in log_10_ concentrations in surface and reclaimed water was statistically significant (*p*-value: < 0.01), but for *Giardia*, the difference was not statistically significant (*p*-value: 0.09).

### 2.2. Indicator Pathogen Correlation

Surface water samples (n = 22) were analyzed by treatment plant (n = 5) and as a pooled data set (all facilities) to determine if the concentrations of the indicators (total coliforms, *E. coli*, *Enterococcus* spp., pasteurized and unpasteurized *C. perfringens*, somatic, F+, and total coliphages) were correlated with each other or with the concentrations of the pathogens (*Salmonella* spp., *Cryptosporidium*, *Giardia*, and Adenovirus A–F) (Table 1). Norovirus GII was not included in the analysis because it was not detected in any of the surface water samples.

**Figure 1:**
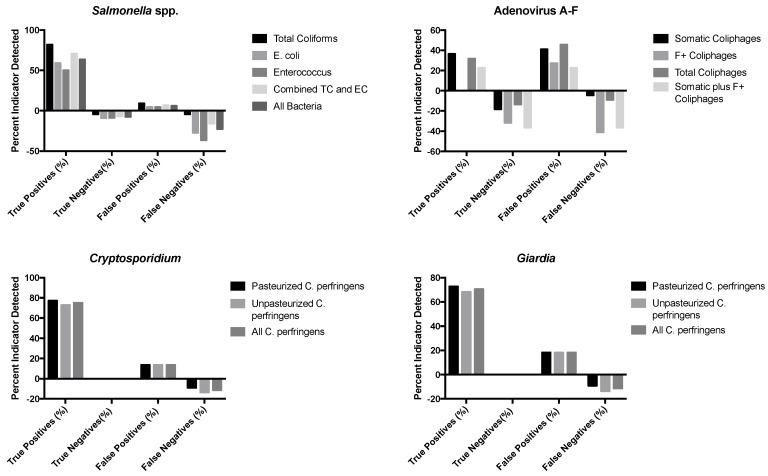
Associations between detection of indicators and combination of indicators and pathogens detected in sewage-impacted surface waters. Percentages were calculated from the total sample number (n = 22).

No significant correlations were found in the analysis of results within facilities. This may be due to the small sample size. However, significant correlations were found between pooled data sets of the log_10_ concentrations of *Salmonella* spp. and total coliforms (Spearman’s *r_s_* = 0.51; *p* = 0.02) and between Adenovirus groups A–F and F+ coliphages (Spearman’s *r_s_* = −0.43; *p* = 0.05). Figure 1 displays the binary logistic regression analysis between *Salmonella* spp. and total coliform bacteria. Significant correlations were also observed between the concentrations of indicator organisms in the pooled data sets; specifically, these included the correlation between the concentrations of *Enterococcus* spp. and *E. coli* (Spearman’s *r_s_* = 0.68, *p* ≤ 0.01), somatic and F+ coliphages (Spearman’s *r_s_* = 0.58, *p* ≤ 0.01), somatic and total coliphages (Spearman’s *r_s_* = 0.94, *p* ≤ 0.0001), F+ and total coliphages (Spearman’s *r_s_* = 0.58, *p* ≤ 0.01), and pasteurized and unpasteurized *C. perfringens* (Spearman’s *r_s_* = 0.98, *p* ≤ 0.0001).

Adenoviruses were found above detection limits in 41% of the surface water samples (n = 22); coliphage viruses co-occurred with adenovirus in 78% of these samples for total coliphages and 67% for somatic coliphages. There was no adenovirus co-occurrence for F+ coliphages. *Cryptosporidium* oocysts were present and above the detection limits in 86% of samples and co-occurred with both pasteurized and unpasteurized *C. perfringens* in 100% of samples examined. Similarly, *Giardia* cysts were detectable in 81% of samples and there was a co-occurrence of 100% with both pasteurized and unpasteurized *C. perfringens*.

Binary logistic regression analysis was also used to test the hypothesis that indicator organisms were correlated with the presence or absence of pathogens in sewage–impacted surface waters. The data for the detected pathogens (*Cryptosporidium*, *Giardia*, *Salmonella* spp., and adenoviruses) were converted to binary data, either pathogen present (1) or pathogen absent (0), and compared to the detected concentrations of their respective fecal indicators (total coliforms, *E. coli*, and *enterococci* as bacteria indicators, *C. perfringens* as protozoan parasite indicators, and the different coliphages (somatic, male-specific/F+, and total) as virus indicators), with evaluations for the relationships between and among the two groups of microorganisms based on presence or absence in samples. Nagelkerke’s R-square, which ranges from 0.0 to 1.0, indicates the strength of the association; stronger associations have values closer to 1.0. An indicator–pathogen combination that displayed a moderate correlation was F+ coliphages and adenovirus presence/absence, with an R-square of 0.48. A much stronger association was seen between *E. coli* and *enterococcus* spp. as fecal indicator bacteria (R-square = 0.71) and between pasteurized and unpasteurized *C. perfringens* as protozoan parasite indicators (R-square = 0.77).

Figure 1 displays the results of this binary logistic regression analysis. True positives were positive for fecal indicators and pathogens, true negatives were samples negative for both fecal indicators and pathogens, false positives were positive for the indicator but negative for the pathogen, and false negatives were positive for the pathogen and negative for the indicator. The sum of each of these categories is 100% for each indicator–pathogen grouping. For many of the fecal indicator organisms evaluated here, especially the fecal indicator bacteria for *Salmonella* and *Clostridium perfringens* for the two protozoan parasites, *Cryptosporidium* and *Giardia*, there was a high true positive rate, typically of about 50% or more for the FIBs and about 70% or more for *C. perfringens*, indicating that the pathogen and the indicator were both present and co-occurred in the surface water. However, there was often not a correspondingly high true negative rate for many of these indicators, including *C. perfringens* and the FIBs. For the viruses, the fecal indicator viruses (somatic, male-specific/F+, and total coliphages) gave true positive and true negative rates that were in the range of about 20–35% and 10–35%, respectively. However, there were also relatively high rates of false positives (about 20 to 40%) and sometimes false negatives (about 40% for both male-specific/F+ and total coliphages). There were no true positives for Adenovirus A–F detected using the male-specific/F+ coliphage indicator.

## 3. Discussion

Pathogens were detected in nearly all surface waters. *Salmonella* spp. was found in 91% of all samples at concentrations from 0.1 to 1.2 MPN/100 mL. Adenoviruses (as detected by molecular methods) were found in 41% of all samples at concentrations ranging from 1 to 3.60 × 10^4^ GECs/100 mL. *Cryptosporidium* and *Giardia,* as detected using US EPA Method 1623, were found in 100% and 81% of all samples, respectively. Total coliforms, *E. coli*, and *Enterococcus* were detected in 95%, 64%, and 50% of samples (respectively), while somatic, F+, and total coliphage viruses were detected in 77%, 32%, and 77% of samples, respectively. *C. perfringens’* spores and vegetative cells plus spores were detected in 91% of all samples. As pathogen and indicator analyses were for different volumes of surface water (10 L for pathogens and 100 mL for indicators), it is likely that the different sample volume for pathogens and indicators impacted the detectability of each microorganism. In general, a larger sample volume and sample size is desirable and would improve pathogen detection.

A weak but statistically significant relationship was found by binary logistic regression between the presence or absence of adenoviruses and F+ coliphages, but this was a negative relationship when adenoviruses were present and F+ coliphages were absent. Additionally, the log_10_ concentration of adenoviruses was negatively correlated with the log_10_ concentration of F+ coliphages by Spearman’s correlation analysis. It is important to note that low levels of F+ coliphages were detected in the surface water samples by a culture method, while relatively high levels of genome-equivalent copies (GEC) of adenovirus were detected by a nucleic acid amplification method. Despite the apparent correlations, levels of the indicator organisms (F+ coliphages) were neither higher than nor positively associated with the pathogen in this case. This is not an ideal quality of an indicator organism and is not necessarily protective of human health for surface water systems. As adenoviruses were detected by qPCR methods but not infectivity, an important caveat in the evaluation of this indicator–pathogen relationship is the absence of infectivity data for these pathogenic viruses as well as their undocumented survival in surface waters. It was shown previously in reclaimed water studies that the ratio of adenovirus gene copies to infectious adenoviruses averages 204 gene copies per 1 infectious unit and ranges from about 25 to 550 gene copies per infectious unit [15].

The binary logistic regression analysis showed that indicator presence or absence was not consistently predictive of pathogen presence, and the results indicated a high number of false-negative or false-positive values for one of the indicator pathogen combinations, specifically the adenovirus/F+ coliphage relationship. Those indicators that were detected more frequently, such as F+ coliphages, showed a higher frequency of false positives (pathogens absent, indictors present). As the goal of an indicator microorganism is to trigger an alert for pathogen presence, rather than for pathogens to be present at equal or greater numbers than the indicator, this is not necessarily an undesirable result. Pathogens detected less frequently, such as *Salmonella* spp., showed a higher frequency of true positives (pathogens present, indicators present). As *Salmonella* was detected at concentrations on average 100-fold lower than the indicator organism, these results represent an ideal indicator organism relationship. FIB occurrence was not predictive of *Salmonella* spp. presence by binary logistic regression, but *Salmonella* spp. was statistically significantly correlated with the concentrations of total coliform by Spearman’s correlation analysis. As such, these results suggest that there may not be one “ideal” indicator for the prediction of survival or presence of pathogens in surface water even when there is evidence that log_10_ concentrations of indicator organisms are often correlated with pathogen concentrations.

Although individual indicator organisms and pathogens were weakly correlated or uncorrelated by binary logistic regression, the data in Figure 1 displays evidence that log_10_ concentrations of indicator organisms are correlated with log_10_ concentrations of pathogens in surface water. This suggests that enteric pathogens, including *Salmonella* bacteria, human enteric viruses such as Adenoviruses and the protozoan parasites *Cryptosporidium* and *Giardia*, are often present at detectable concentrations in surface waters that may be used as drinking water sources. In the analysis of source waters for which de facto reuse occurs, such as those we observed in North Carolina, the use of surface waters currently available would result in the use of a lower quality of water than what is produced by advanced wastewater treatment for reuse.

As US EPA Method 1623 does not allow for the determination of (oo)cyst infectivity or the detection of human specific (oo)cysts, these are important limitations to this study, especially the lack of infectivity data on the protozoan parasites [16]. In this study, *Cryptosporidium* and *Giardia* were found at low levels by immunofluorescent microscopy in nearly all surface water samples, but infectivity was not assessed due to the lack of time and additional resources needed to process and perform *Cryptosporidium* infectivity analyses for these surface water samples. Although the presence of these pathogens in surface water is of some concern, it is difficult to evaluate the human health risk posed by these microorganisms in the absence of infectivity data for them. In studies where infectivity was evaluated using US EPA method 1623, typically approximately 20% of the *Cryptosporidium* detected were viable [17].

One of the primary findings of this research is that no one indicator is ideal for the prediction of survival or presence of pathogens in surface water. Practically, this means that in public health and wastewater management programs and practices, it is important to test for a variety of microorganisms using multiple methods to more reliably and accurately determine quality. Other studies evaluating the relationship between indicators and pathogens, particularly enteric viruses and coliphages, have also found differences in the survival of these microorganisms in surface waters [18,19,20]. Proposed reasons for these differences include morphological differences as well as ssRNA vs. dsDNA genomes in different viruses [21].

Limitations of the current study also include the small number of samples (n = 22), from a limited number of sample sites (n = 5). Additional studies are needed to evaluate more thoroughly and rigorously the relationships between the fecal indicators and the enteric pathogens in these surface waters. Consideration should also be given to questions of infectivity and culturability of enteric viruses and protozoan parasites in order to evaluate more accurately the human health risk from these pathogens in surface water samples used for beneficial purposes, including primary contact recreation and potable reuse.

## 4. Materials and Methods

### 4.1. Sample Collection and Storage

Surface water samples were collected from two run of river drinking water treatment plants and two sewage-impacted reservoir source drinking water treatment plants in central North Carolina. Sewage-impacted reservoirs were influenced by runoff or discharge from a wastewater treatment facility. The facilities included a total of five sample collection points: three treatment facilities with drinking water reservoirs (1) the Cary/Apex Drinking Water Treatment Plant, using Jordan Lake; (2) the E.M. Johnson Water Treatment Plant, using Falls Lake; and (3) the Smithfield Water Treatment Plant using the local reservoir. The study also included two run of river drinking water treatment plants: (1) the Hillsborough Drinking Water Treatment Plant, using the Eno River, and (2) the Smithfield Water Treatment Plant using Neuse River. Surface waters were collected as grab samples in sterile bottles and kept chilled in coolers with ice during transport to the laboratory in Chapel Hill. Samples collected from treatment plants with reservoirs (Cary/Apex, and E.M. Johnson) were collected from the water treatment plant intake structure. Run of river treatment plant samples and the Smithfield Reservoir samples were collected approximately 2 m from shore and approximately 1 m below the surface of the water. The samples were stored at 4 °C upon arrival at the laboratory.

### 4.2. Sample Processing and Microbial Detection

**Figure 2:**
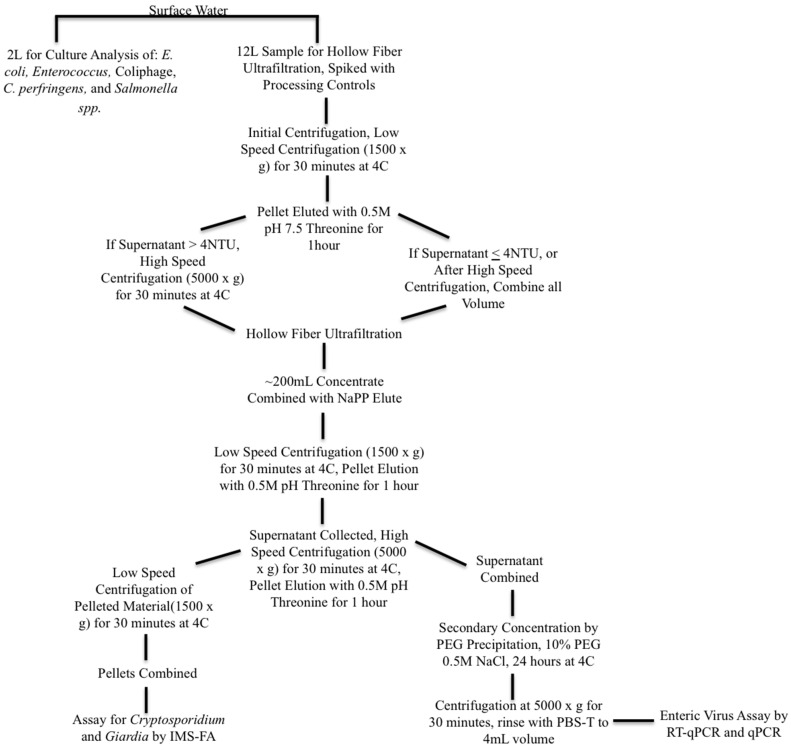
Diagram of surface water sample processing for indicator and pathogenic microorganisms. Legend for Figure abbreviations: PEG: polyethylene glycol, NAPP: Sodium polyphosphate, NaCl: Sodium chloride, PBS-T: phosphate buffered saline with Tween-80, IMS-FA: immunomagnetic separation florescent antibody staining.

Surface water samples were collected as 16-L volumes and split into a 12-L volume for pathogen analysis and a 4-L volume for indicator analysis. Samples were processed and concentrated according to the procedures described in Bailey et al. (2018) [15], with the addition of an initial centrifugation of 1500× *g* for 30 min as a step applied to the sample volume for the enteric virus concentration method in order to remove sediment and other solids before hollow fiber ultrafiltration. If the supernatant turbidity was greater than 4 NTU (a turbidity appropriate for hollow fiber ultrafiltration), the surface water was centrifuged again at 5000× *g* for an additional 30 min. Viruses in the centrifuged sediment were recovered by elution at 60 RPM with five parts 0.5 M, pH 7.5 Threonine to one part surface water solids for 1 h, and added back to the concentrated supernatant for further processing and analysis, following the method of Shieh et al. (1997) [22]. Sample processing and concentration steps for surface water are summarized in Figure 2. Methods for the detection of pathogenic and indicator organisms are as described in Bailey et al. (2018) [15].

### 4.3. Statistical Analysis

In order to evaluate the relationship between indicator organisms and pathogens in surface water samples, the detected concentrations were first log_10_ transformed and analyzed using GraphPad Prism 7 (GraphPad Software, Inc., La Jolla, CA, USA). An ANOVA regression analysis was performed using a Tukey posttest on log_10_ concentration data, which uses group means to compare differences among surface water samples. Specifically, the mean log_10_ concentration of each indicator organism was compared with each of the other indicator organisms by class and also with the relevant pathogen detected. To evaluate the correlation between indicators and pathogens in these samples, Pearson’s test was used for normally distributed data or relevant nonparametric tests were used for data not normally distributed. The purpose of this test is to measure the linear dependence or correlation between two variables by using linear regression tools. In this analysis, indicator organisms were evaluated for their correlative relationship to other indicators and pathogens. Additionally, a binary logistic model was used to test the hypothesis that indicator organism concentrations were predictive of the presence or absence of pathogens in surface water, as described by Harwood et al. (2005) [17]. Briefly, this method involved the use of continuous independent variables with non-detectable values being reported as a value of 0. The data for indicator organisms (total coliforms, *E. coli*, *C. perfringens*, and coliphages) were then converted into a string of binary variables that represented the presence or absence of each indicator. The ability of the indicator data string to predict the presence of each pathogen (*Cryptosporidium*, *Giardia*, *Salmonella* spp., adenoviruses, and noroviruses) was assessed separately and also for all viruses (Adenovirus groups A–F, Norovirus GII, and combined as an enteric viruses category). Results were expressed as the percentage of samples correctly classified into the “pathogen present” and “pathogen absent” categories. Binary logistical modeling was conducted using SPSS Version 24 (IBM Corporation, Armonk, NY, USA).

## 5. Conclusions

The primary finding of this research was that no one indicator was perfect in the prediction of survival or presence of pathogens in surface water. For public health and wastewater management programs and practices, it is important to test for a variety of microorganisms using multiple methods to accurately determine water quality. Consideration should also be given to questions of infectivity and capturability of enteric viruses and protozoan parasites to estimate human health risk from these pathogens in waters used for primary contact recreation or drinking water purposes.

## Figures and Tables

**Table 1:** Statistical comparisons of indicator and pathogen concentrations in water samples (n = 22).

Concentration Analysis				
Organism 1	Organism 2	Mean Difference	*p*-value	Significant?
Total Coliforms	*E. coli*	2.17	<0.0001	Y
*E. coli*	*Enterococcus*	0.20	0.51	N
*Enterococcus*	Total Coliforms	2.37	<0.0001	Y
*Salmonella*	Total Coliforms	−3.20	<0.0001	Y
	*E. coli*	−1.03	<0.01	Y
	*Enterococcus*	−0.84	0.01	Y
Somatic Coliphage	F+ Coliphage	0.79	<0.01	Y
F+ Coliphage	Total Coliphage	−0.81	<0.01	Y
Total Coliphage	Somatic Coliphage	−0.03	1.00	N
Adenovirus A–F	Somatic Coliphage	2.80	<0.0001	Y
	F+ Coliphage	3.58	<0.0001	Y
	Total Coliphage	2.77	<0.0001	Y
Total *C. perfringens*	*C. perfringens* spores	0.04	1.00	N
*Cryptosporidium*	Total *C. perfringens*	−1.74	<0.0001	Y
	*C. perfringens* spores	−1.78	<0.0001	Y
*Giardia*	Total *C. perfringens*	−2.35	<0.0001	Y
	*C. perfringens* spores	−2.36	<0.0001	Y
*Cryptosporidium*	*Giardia*	0.60	<0.01	Y

## Data Availability

Data are available upon reasonable request from the corresponding author.
